# Convolutional Neural Network-Based Shadow Detection in Images Using Visible Light Camera Sensor

**DOI:** 10.3390/s18040960

**Published:** 2018-03-23

**Authors:** Dong Seop Kim, Muhammad Arsalan, Kang Ryoung Park

**Affiliations:** Division of Electronics and Electrical Engineering, Dongguk University, 30 Pildong-Ro 1-Gil, Jung-Gu, Seoul 100-715, Korea; k_ds1028@naver.com (D.S.K.); arsal@dongguk.edu (M.A.)

**Keywords:** intelligence surveillance camera, shadow detection, color feature, CNN

## Abstract

Recent developments in intelligence surveillance camera systems have enabled more research on the detection, tracking, and recognition of humans. Such systems typically use visible light cameras and images, in which shadows make it difficult to detect and recognize the exact human area. Near-infrared (NIR) light cameras and thermal cameras are used to mitigate this problem. However, such instruments require a separate NIR illuminator, or are prohibitively expensive. Existing research on shadow detection in images captured by visible light cameras have utilized object and shadow color features for detection. Unfortunately, various environmental factors such as illumination change and brightness of background cause detection to be a difficult task. To overcome this problem, we propose a convolutional neural network-based shadow detection method. Experimental results with a database built from various outdoor surveillance camera environments, and from the context-aware vision using image-based active recognition (CAVIAR) open database, show that our method outperforms previous works.

## 1. Introduction

Because the detection of moving objects is demanded in various areas, including surveillance camera system functions, it is a very important research subject in computer vision. Surveillance camera systems use the background subtraction operation, which detects the foreground to detect a moving object. However, various environmental factors such as illumination change and brightness of background cause the precise foreground detection to be a very difficult task. Particularly, the shadow is a typical barrier that makes exact detection of foreground and recognition of objects difficult [1,2,3,4,5,6]. The detection error for a shadow may cause an object to be identified as a larger object. In the real-time surveillance system for an outside environment, this error can cause a man to be mistaken for a vehicle. Moreover, the shadow detection error causes another problem related to human detection, because multiple people can be detected as one human. This is because size information is a key factor in detecting and recognizing humans. Additionally, the effective removal of shadow is essential to template-matching, histogram-matching, and other object detection algorithm functions. Existing methods of detecting and removing shadow use shadow chromaticity of various color spaces, or add further information (e.g., gradient), and then utilize reference values trained to detect shadow. Further details are presented in Section 2.

In this study, we propose a convolutional neural network (CNN)-based shadow detection method, and our research is novel in the following four ways, compared to previous works.

1. This is the first CNN-based approach to shadow detection.
2. We convert input image of red-green-blue (RGB) color into that of hue-saturation-value (HSV) coordinate to remove the effect of hue channel which causes the error of shadow classification. As the input to CNN, we use an image of three channels including the saturation and value images of input, and the ratio image of value images of input to background.
3. The searching region including a rough area of foreground and shadow is determined by background subtraction. To reduce the processing time, only the 21 × 21 sliding window extracted from this searching region is used for the input to VGG Net-16 model.
4. We open our CNN model trained in this research and the experimental database in [7], so that other researchers can perform a fair comparison.

## 2. Related Works

There are various methods of shadow detection. In this research, we classify those methods into non-learning-based and learning-based methods by referring to [2]. Section 2.1 explains non-learning-based shadow detection algorithms using color and other information in various color spaces. Section 2.2 explains learning-based algorithms for detecting shadow.

### 2.1. Non-Learning-Based Methods

Various algorithms have been proposed to detect and remove shadow. Among them, the algorithm using shadow color information has been most widely applied [8,9,10,11,12,13,14]. Shadow brightness generally decreases in the background but does not change its chromaticity value. This characteristic of shadow is used to detect shadows in various color spaces, including hue, saturation, and value (HSV) [8], red, green, and blue (RGB) [9], *C*_1_*C*_2_*C*_3_ [10], normalized RGB [11,12], luma and chrominance (YUV) [13], and luma, blue-difference, and red-difference (YCbCr) [14]. The research selected HSV to detect shadow considering the color perception of human [8]. Because a shadow darkens the background, whereas the foreground varies depending on its color, HSV values were compared between the background and each pixel of the input image to detect and remove shadow. The research of [9] proposed an algorithm that calculates brightness and chromaticity in RGB color space and uses the calculations to detect shadow. The shadow is then identified as a region where the chromaticity is similar, whereas the brightness is lower than background. The research of [10] selected the *C*_1_*C*_2_*C*_3_ color space for shadow detection. Along with the photometric color invariant property of shadow, the geometrical property was also considered, which is the boundary property of shadow appearing alongside light and beside an object. Because the conventional RGB color space is very sensitive to light, the normalized RGB was used to minimize the light’s impact [11,12]. In this research, we also conduct normalization by dividing each RGB value by the sum of pixel values of each RGB. The research of [13] used the YUV color space used in conventional TV, image, and video encoding. Whereas other research required transformation into HSV or another color space to create a system similar to the perceived color space of a human, the research of [13] used YUV color space to remove the processing time needed for the transformation. Thus, it obtained approximated values of color change, which, however, were not absolute values of hue and saturation. The research of [14] selected the YCbCr color space for shadow detection. Y is luminance, and Cb and Cr indicate color information. The Y value was used for shadow detection. The information of Y channel was used to identify candidate shadow regions. Then, the sliding window was applied to detect shadow.

Most of the above research used simple color or Y information. Therefore, if an object had a similar color to shadow, accurate detection was expected. Thus, the research did not produce robust results for more varied data. To solve this problem, other researchers have used additional shadow properties and information. The research of [15] applied gradient information with color information for shadow detection. Color information is used in HSV color space to obtain a candidate color region. The candidate region is a large region including, ideally, the entire shadow. In this case, after the candidate region was detected, the gradient magnitude and the gradient direction were used to distinguish the foreground, background, and shadow. The research of [16] also utilized the generic properties of shadow and applied all the properties proposed by the other studies. Luminance, chrominance, the difference in gradient density between shadow and background, and the boundary characteristics of foreground were used to calculate a shadow confidence score in a candidate shadow region. This score was then used to detect shadow. The research of [17] detected a candidate shadow region under the assumption that the shadow region in gray images is half-transparent and has a similar value to the corresponding region of the background. Thus, the Gabor filter, which is usually applied to a small region, was used to extract features, and finally detect the shadow. Such shadow detection at a region level is more robust than the shadow detection at a pixel level. The research [3] proposed the method of shadow detection-based foreground detection, vertical histogram analysis, foreground partitioning, calculation of the orientation of major axis, and decision, but it used the assumption that the position of light source should be known in advance and the light source should not exist at the upper position of pedestrian.

Because every research mentioned above mainly used only shadow color or gray texture information, they were susceptible to changes in objects and illuminators, which have similarities to shadow color and gray texture. All the above methods proposed by the existing research are limited in their application to real-world environments containing lots of variables.

### 2.2. Learning-Based Methods

The learning-based shadow detection methods were proposed to solve the disadvantages of the non-learning-based methods, which are explained in Section 2.1. In previous research [18,19,20], the Gaussian shadow model or the Gaussian mixture model (GMM) was applied to design a statistical model of shadow properties. For segmentation of shadow, the research of [18] used the attenuation ratio of luminance and chrominance of shadow surfaces in YUV color space. The research of [20] simulated shadow and background by using physics-based color features. GMM, which is based on spectral ratio and gradient intensity distortion, was used to learn shadow models and to then detect shadow. The research of [19] utilized geometrical properties of shadow and human regions. A rough shadow region was initially detected, and a Gaussian shadow modeling was conducted by using the center of gravity and the orientation of the detected region. In [21], shadow was detected by statistical modeling based on a hidden Markov model (HMM). From the histograms of many shadow, foreground, and background images, the average and deviation of each region were obtained. Then, each region was modeled using an independent HMM for shadow detection. The research of [22] proposed a shadow detection algorithm using a neural-fuzzy system. Based on color features obtained in HSV color space, a self-organizing map with a fuzzy inference Sugeno system was used for shadow detection. The research of [23] proposed a shadow detection method using the principal component analysis (PCA) and GMM algorithm. GMM generated a background image from the input image, and PCA extracted features of the input image and background image. Then, the shadow was detected through a Euclidean distance. The research of [24] presented a shadow detection algorithm applying a support vector machine (SVM) with a Gaussian kernel. Chromaticity, intensity, and edge were used as learning features.

These learning-based methods showed better performance than non-learning-based methods, but they could not manually extract optimal hand-craft features and were applicable only to specific environments. Consequently, the learning-based methods cannot be applied to various environment types for shadow detection. To solve this problem, our research proposes a convolutional neural network (CNN)-based shadow detection method. Table 1 shows the summarized comparisons of previous and proposed methods on shadow detection.

The remainder of this paper proceeds as follows. Section 3 introduces the proposed CNN-based shadow detection method; Section 4 presents experimental results and analysis; and Section 5 summarizes the conclusions of this research.

## 3. Proposed Method

### 3.1. Overall Procedure of Proposed Method

**Figure 1:**
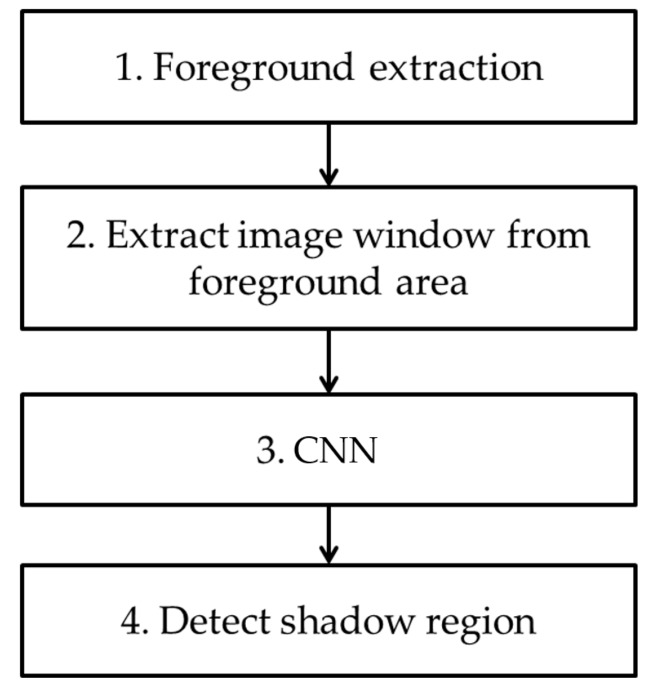
Overall flowchart of proposed method.

In Figure 1, we show the overall flowchart of our method. In the first step, a foreground region is detected through background subtraction using the background image. Then, a window image with a 21 × 21-pixel size is extracted from the detected foreground region. After the extracted window image is resized to a 224 × 224-pixel size, it is input into a pre-trained CNN. A shadow region is then detected based on the output of CNN. In this research, we use VGG Net-16, which is pre-trained with ImageNet dataset [25,26], as CNN. Further fine-tuning with the experimental data used in this research is conducted before testing.

### 3.2. Extraction of Window Image for CNN Input

**Figure 2:**
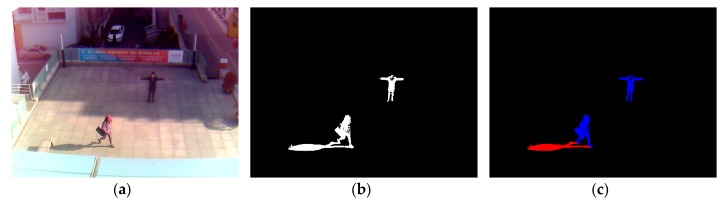
Example of input image, the result of foreground detection, and ground-truth image of non-shadow (human) and shadow areas. (**a**) Input image; (**b**) Result of foreground detection; (**c**) Ground-truth image of non-shadow (blue color) and shadow (red color) areas.

After obtaining a foreground region via background subtraction, as shown in Figure 2b, non-shadow (i.e., human) and shadow areas are manually separated as ground-truth regions for CNN training, as shown in Figure 2c. That is, the shadow pixels among the white pixels of Figure 2b are manually painted as red color by the observation of human developer, and the remaining white pixels are automatically converted into those of blue color as shown in Figure 2c. This is ground-truth data, and it is used for CNN training and measuring the accuracy of shadow detection in our experiment. In detail, based on the positions of ground-truth regions, the window images of 21 × 21 pixels are extracted from the original input image of Figure 2a. For example, the window whose center belongs to the blue region of Figure 2c is determined as a non-shadow area. Whereas, the window whose center belongs to the red region of Figure 2c is determined as a shadow area. The window image of 21 × 21 pixels is extracted from the input image of HSV color space instead of RGB color space.

The existing researches have assumed that shadow darkens the background but retains its color, whereas the chromaticity of a human figure shows a more diverse change against the background [8,15]. Based on the result of [8], we experimentally determine that the HSV color space is the most suitable for representing shadow features. Accordingly, input and background images are transformed into an HSV color space. If the input and background images transformed from RGB color space to HSV color space are *I* and *B*, respectively, each channel value of the window image $F_{k,i}\left(x,\;y\right)$ created is defined by Equation (1).

(1) $$F_{k,i}\left(x,\;y\right)=\{\begin{array}{c} F_{k}^{1}\left(x,\;y\right)=I_{k}^{s}\left(x,\;y\right)\\ F_{k}^{2}\left(x,\;y\right)=I_{k}^{v}\left(x,\;y\right)\\ F_{k}^{3}\left(x,\;y\right)=\frac{I_{k}^{v}\left(x,\;y\right)}{B_{k}^{v}\left(x,\;y\right)} \end{array}$$

where, $F_{k,i}\left(x\;,y\right)$ is the *i*th window image in the *k*th image frame, and $F_{k}^{1}\left(x,\;y\right),\;F_{k}^{2}\left(x,\;y\right),\;F_{k}^{3}\left(x,\;y\right)$ are each a channel image of $F_{k,i}\left(x\;,y\right)$. *I_k_*(x, y) and *B_k_*(x, y) are the input image and the background image, respectively. *s* is saturation and *v* is intensity of the image in HSV. The size of generated window image is 21 × 21 × 3 (i.e., width × height × channel), and the extraction uses *x*, *y* nominal coordinates. Window images extracted from training data are used for CNN training. As shown in Figure 2b, the window image extracted from testing data through background subtraction is used as input into CNN. By this method, non-shadow and shadow regions are distinguished from each other.

### 3.3. VGG Network for Classifying Non-Shadow and Shadow Regions

**Figure 3:**
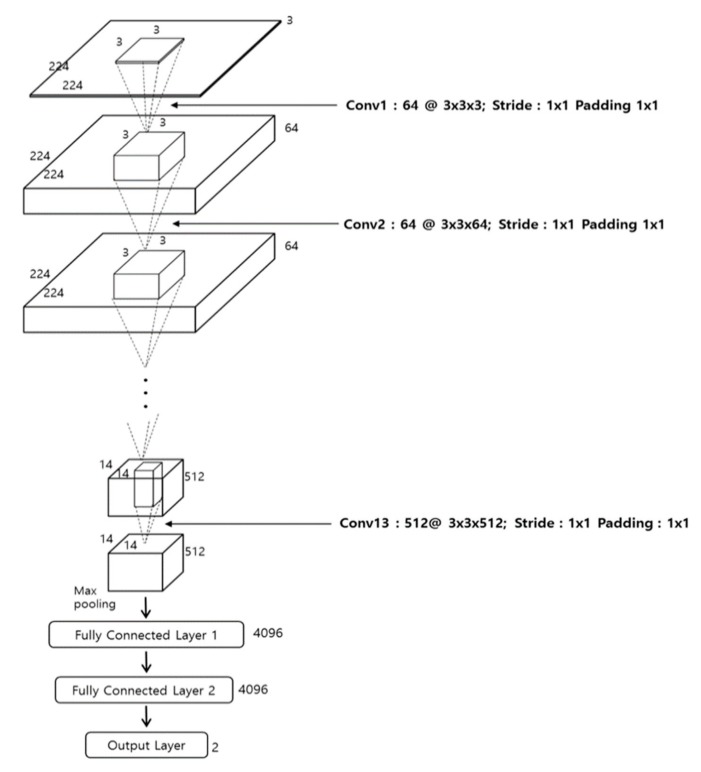
VGG Net-16 architecture used in our research.

Figure 3 and Table 2 show the overall architecture of the VGG Net-16 [25] used in this research. The VGG Net-16 consists of a total of 16 layers, including 13 convolutional layers and three fully connected layers (FCL). Every convolution layer is connected to the rectified linear unit (ReLU) layer. We apply fine-tuning to the network, which is pre-trained through the ImageNet dataset [27,28]. Further details are explained in Section 4.

The initial image size of this CNN is 224×224×3. Accordingly, as mentioned in Section 3.2, we conduct bilinear interpolation to resize the three-channel 21 × 21 window image, which is obtained from HSV color space, into the three-channel 224×224 image, which is used as the input of CNN.

In the first convolutional layer, 64 filters with of size 3 × 3 × 3 are used. The feature map size is 224 × 224 × 64 in the first convolutional layer, such that 224 and 224 are the output height and width, respectively, calculated based on (output height (or width) = (input height (or width) − filter height (width) + 2 × (the number of padding))/(the number of stride) + 1 [29]). For example, because the input height, the filter height, the number of paddings, and the number of strides in the image input layer and first convolutional layer in Table 2 are 224, 3, 1, and 1, respectively, the output height becomes 224 = (224 − 3 + 2 × 1)/1 + 1.

The output feature map for standard convolution based on stride one and padding is usually obtained as [30]:

OF*_k,l,n_* = Σ*_i,j,m_* (K*_i,j,m,n_* ⋅ IF_*k+i*−1,*l+j*−1,*m*_) (2)

In Equation (2), IF*_k+i_*_−1*,l+j*−1*,m*_ is the input feature map of size, *A_F_* · *A_F_* · *U*. *A_F_* is the width and height of square input feature map, and *U* is the number of input channels (i.e., input depth). OF*_k,l,n_* is the output feature map of size, *B_F_* · *B_F_* · *V*. *B_F_* is the spatial width and height of a square output feature map, and *V* is the number of output channels (i.e., output depth). In Equation (2), K*_i,j,m,n_* is the convolution kernel of size, *A_K_* · *A_K_* · *U* · *V*, and *A_K_* is the spatial dimension of the convolution kernel. Then, standard convolutions take the following computational cost.

*C* = *A_K_* · *A_K_* · *U* · *V*·*A_F_* · *A_F_* (3)

Based on Equation (2), we find that the computational cost is dependent on multiplicatively on the kernel size, *A_K_* · *A_K_*, the number of input channels of *U*, the number of output channels of *V*, and the input feature map size, *A_F_* · *A_F_* [30].

The image is calculated by the above equation and the result is input into the next layer. As shown in Equation (4), every convolution layer is connected to the ReLU layer, which has non-saturating nonlinearity, and is faster than an activation function with saturating nonlinearity. For example, *f*(*x*) = *tanh*(x) and *f*(*x*) = ${\left(1+e^{-x}\right)}^{-1}$, etc. thus, it can remove the vanishing gradient problem in the back propagation at the time of training [31,32]. In [27], they showed that the speed of training by ReLU with the CIFAR-10 dataset, based on the four-layered CNN, is six times faster than the *tanh*(x) function with same dataset and network.

(4) y=max(0,x),

where *x* and *y* are input and output values of the ReLU function, respectively. As shown in Table 2, the feature map obtained by conducting ReLU after the first convolutional layer passes through the max pooling layer after the second convolutional layer and another ReLU. Here, as with the first convolutional layer, the second applies the same filter height and height of 3 × 3, a padding of 1 × 1 and a stride of 1 × 1, and retains a feature map size of 224 × 224 × 64. It is clear in Table 2 that 13 convolutional layers commonly use the filter size (i.e., width and height) of 3 × 3 and the padding of 1 × 1, thereby retaining the feature map size (i.e., width and height). Only the number of filters is changed to 64, 128, 256, and 512. Each ReLU layer is connected to the back of each convolutional layer, and the feature map size is retained after passing through convolutional layers. After the 2nd, 4th, 7th, 10th, and 13th convolutional layers with ReLU, a max pooling layer is connected. The max pooling layer uses the maximum value within a filter of a specified size and performs a type of subsampling work.

After the second convolutional layer with ReLU, when the max pooling layer operates, the input feature map size is 224 × 224 × 64, the filter size is 2 × 2, and the number of stride is 2 × 2. When the number of stride is 2 × 2, it implies a max pooling filter of 2 × 2. That is, there are pixel movements in horizontal and vertical directions. Because there is no overlapped area during the movement of filters, the feature map size is reduced to 1/4 (i.e., 1/2 in width and 1/2 in height). Ultimately, the feature map size, which passes the max pooling layer, becomes 112 × 112 × 64 pixels. As in Table 2, this max pooling layer consists of a filter of 2 × 2 and a stride of 2 × 2 in every case. For this reason, the feature map size is reduced to 1/4 (i.e., 1/2 in width and 1/2 in height). After passing through 13 convolutional layers, 13 ReLU layers and 5 max pooling layers, the final feature map size becomes 7 × 7 × 512 pixels, and the map passes through an additional three FCLs. The output nodes of the first, second, and third FCL are 4096, 4096, and 2, respectively.

Generally, CNN has the over-fitting problem, where the network is too dependent on training data. This problem can cause low recognition accuracy with testing data, although the accuracy with the training data is still high. To solve this problem, we use dropout methods [27,33], which can reduce the effects of the over-fitting problem. For the dropout method, we use a dropout probability of 50% to disconnect the previous layer from the next layers in the first and second FCL. After the third FCL, the probability of non-shadow and shadow is calculated by using the SoftMax layer, as shown in Equation (5).

(5) $$\sigma {\left(s\right)}_{j}=\frac{e^{sj}}{\sum_{n=1}^{K}e^{sn}}$$

Given that the array of output neurons is set as *s*, we can obtain the probability of neurons belonging to the *j*th class by dividing the value of the *j*th element by the summation of the values of all the elements. Because only two ultimate classes of non-shadow and shadow exist in this research, the output of classification layer after the third FCL is 2.

## 4. Experimental Results

Training and testing were conducted on a desktop computer with the following specifications: Intel® Core™ i7-6700 CPU @ 3.40 GHz (i.e., 4 cores) [34], 64 GB memory, and the NVIDIA GeForce GTX 1070 graphic card (i.e., 1920 CUDA cores) (NVIDIA, Santa Clara, CA, USA) with 8 GB memory [35]. The CNN training and testing algorithms are implemented with Visual Studio 2013 [36] and Window Caffe (version 1) [37].

### 4.1. Experimental Data

**Figure 4:**
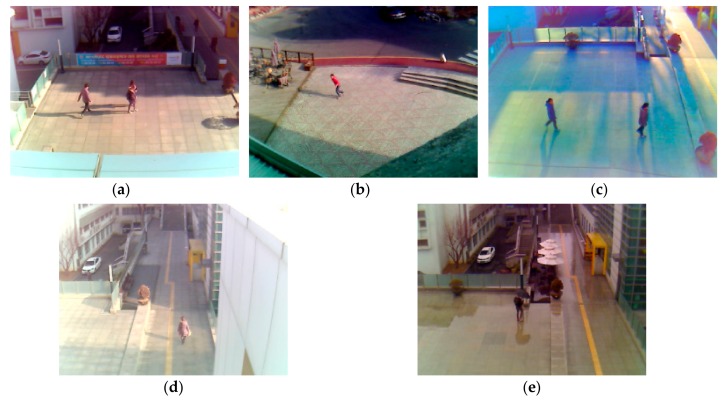
Various images of the experimental database for this research: (**a**) Sub-database 1; (**b**) Sub-database 2; (**c**) Sub-database 3; (**d**) Sub-database 4; and (**e**) Sub-database 5.

The experimental data are obtained by installing visual light cameras 5 to 10 m above the ground [38], which approximates the conventional height of surveillance camera. As shown in Figure 4 and Table 3, images are shot in the morning, the afternoon, the evening, and on rainy days under various weather conditions, temperature, and illumination. A total of 24,000 images, constituting five sub-datasets, are obtained. The original image size is 800 × 600 pixels of the RGB three-channel. For fair comparison to other research, the Dongguk Shadow Detection Database (DSDD-DB1) and the trained CNN model are open to the public in [7].

### 4.2. Training of CNN Model

Window images are extracted from DSDD-DB1, as explained in Section 3.2. The total number of extracted window images is 1008,254 (696,692 non-shadow images and 311,562 shadow images). In this research, we divide our dataset in halves to perform the two-fold cross validation. If those halves are called group 1 and group 2, respectively, as shown in Table 4, then group 1 uses 347,617 non-shadow images and 156,348 shadow images, whereas group 2 uses 349,075 non-shadow images and 155,214 shadow images. In other words, in the first-fold cross validation, the training applies the group 1 data and the testing applies the group 2 data. Alternatively, the second-fold cross validation uses group 2 data for training and group 1 data for testing.

The stochastic gradient descent (SGD) method [39] is used for CNN training. The SGD method is a derivative-based method of finding an optimal weight to minimize the difference between desired output and calculated output. Unlike the gradient descent method, the SGD method defines the division of mini-batch by an iteration of size unit. One epoch is the duration where the iteration number of training is completed. Training is conducted as many as a predetermined number of epochs. In this research, we train CNN by ten epochs. CNN training parameters are as follows. The optimum fine-tuning model is experimentally found, based on the optimal parameters of initial learning rates of 0.001, the momentum value of 0.9, and a mini-batch size of 20. Additionally, the learn-rate-drop is 0.01, and the learning rate decreases by 1/10 of the previous value every 3.3 epochs.

**Figure 5:**
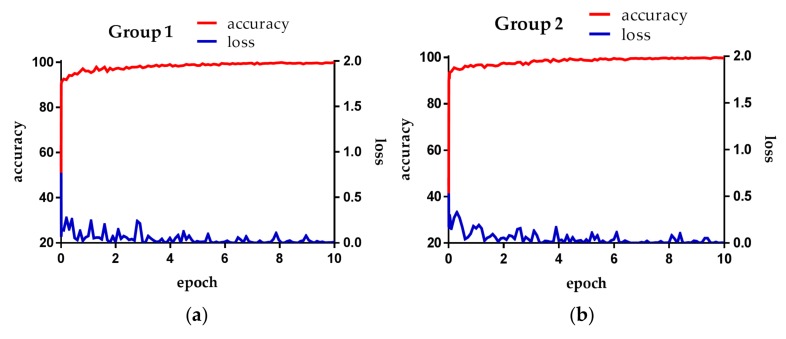
Training loss and accuracy per epoch: (**a**) First-fold cross validation; (**b**) second-fold cross validation.

Figure 5 is the loss and accuracy of each epoch during the training in our experiment. The loss is the training loss, and the accuracy is the degree of training measure. That is, the accuracy obtained by retesting the training data. The loss value depends on learning rate and batch size. If the learning rate is set to a low value, the loss value gradually decreases in a linear form. If the learning rate is high, the loss value decreases drastically and does not reach the desired optimal training result, thereby retaining a loss value.

Figure 5a,b shows loss and accuracy obtained from training in the first- and second-fold cross validations, respectively. Both cases reveal that the increase of training epoch is accompanied by the convergence of loss and accuracy to 0 and 100%, respectively.

**Figure 6:**
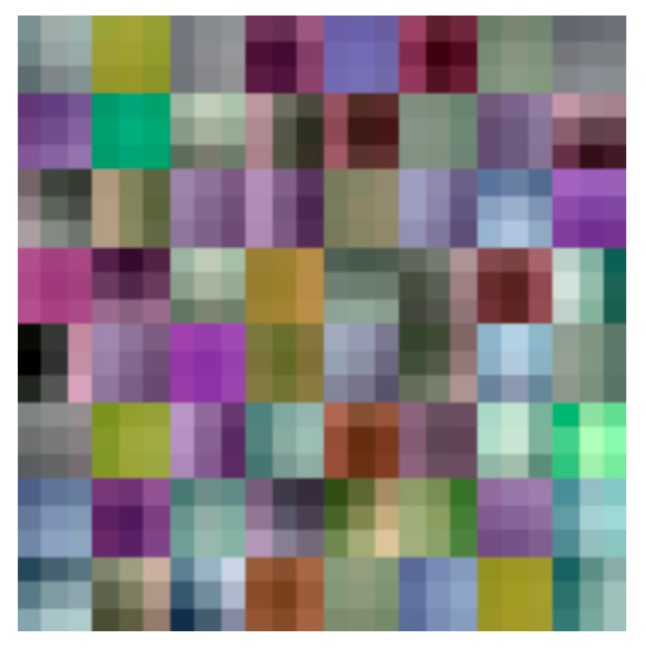
Example of the obtained filters from the first convolutional layer through training.

Figure 6 illustrates the filters in the first convolutional layer of the trained CNN model. As shown in Table 2, the first convolution layer has 64 kernels and the size of 3×3 (i.e., width × height).

### 4.3. Testing of Proposed Method

Table 5 is a confusion matrix showing the testing results. Testing 1 and 2 present the accuracy of testing data for the first- and second-fold cross validations, respectively. If the shadow region corresponds to positive data and the non-shadow region to negative data, the first row, from left to right, indicates the true positive rate (TPR) of identifying shadow correctly and the false negative rate of mistaking shadow as non-shadow. The second row, from left to right, indicates the false positive rate of mistaking non-shadow as shadow and the true negative rate (TNR) of identifying non-shadow correctly.

We measure the testing accuracy by applying Equations (6)–(9), as shown in Table 6. The minimum value and the maximum value are set to 0 and 100, respectively. The higher the value the more accurate. As in Table 5, testing 1 and 2 show the accuracy for testing data in the first- and second-fold cross validations, respectively. In Equations (6)–(9), #TP, #TN, #FP, and #FN indicate the number of true positives (TPs), true negatives (TNs), false positives (FPs), and false negatives (FNs), respectively [40]. As shown in Table 5 and Table 6, the proposed method produces the average shadow detection performance of at least 96%.

(6) $$\mathrm{TPR}=\frac{\#TP}{\#TP+\#FN}$$

(7) $$\mathrm{Positive}\;\mathrm{predictive}\;\mathrm{value}\;(\mathrm{PPV})=\frac{\#\mathrm{TP}}{\#\mathrm{TP}+\#\mathrm{FP}}$$

(8) $$\mathrm{Accuracy}\;(\mathrm{ACC})=\frac{\#\mathrm{TP}+\#\mathrm{TN}}{\#\mathrm{TP}+\#\mathrm{TN}+\#\mathrm{FP}+\#\mathrm{FN}}$$

(9) $$\mathrm{F1}\_\mathrm{score}=2\times \frac{\mathrm{PPV}\times \mathrm{TPR}}{\mathrm{PPV}+\mathrm{TPR}}$$

**Figure 7:**
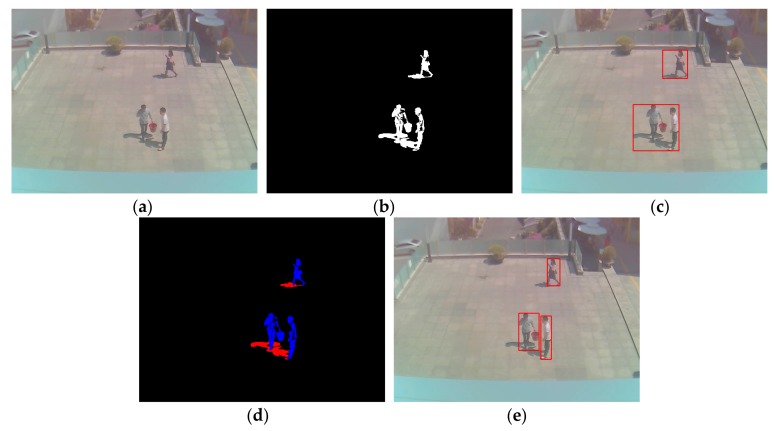
Examples of resultant images of each phase, which are obtained via the proposed method. (**a**) Input image; (**b**) Foreground area obtained by background subtraction; (**c**) Detected box of foreground area; (**d**) Detected shadow (red color) and non-shadow (blue color) regions by our method; (**e**) Final result of non-shadow (human) area excluding shadow region.

Figure 7 presents examples of result images for each phase, which are obtained by the proposed method. As shown in Figure 7, we find that our method detects the correct human area by excluding the shadow region, even with the images of various environments and humans at far distances.

In the next experiment, we compare the performance between the proposed method and the methods [8,15,17,19,20].

As mentioned in Section 2, the method of [8] detects shadow in HSV color space by utilizing the fact that shadows reduce the brightness of the background, whereas its chromaticity does not change much. The method of [15] uses gradient information, along with the existing HSV color information for shadow detection. The method of [17] finds a candidate shadow region under the assumption that the shadow region of a gray image is half-transparent, having a similar value to that of the corresponding background region. The Gabor filter, which is applied to a small region, is used to extract features and to finally detect shadow. The method of [19] uses the geometric properties of shadow and human regions. A rough shadow region is initially detected, and then the orientation and the center of gravity of the detected region are used for the Gaussian shadow modeling of shadow. The method of [20] utilizes physics-based color features to model shadow and background. A shadow model is trained by GMM, based on gradient intensity distortion and spectral ratio, and then shadow is detected.

Additionally, as shown in Table 2, the final two outputs of the classification layer are not used to distinguish shadow and non-shadow, but 4096 features extracted from the first FCL are used to calculate the mean Euclidean distance for each of shadow and non-shadow classes, which are obtained from training data, thereby detecting shadow and non-shadow regions. This scheme is also widely adopted by existing CNN-based recognition research [41]. Besides, apart from VGG Net-16, which is used in this research, AlexNet [27], which has lower depth and CNN architecture, was used to compare performance.

The previous researches [8,15,17,19,20,27,41] have been widely compared for measuring the accuracy of shadow detection in previous works. Except for these researches, there is no more recent method focused on the topic of shadow detection. The methods [5,6] used the method of shadow detection of [8]. In [4], their method of shadow detection was used for detecting only the shadow of building (not pedestrian), and their experimental images were obtained from bird-eye view (like the images captured by airplane) with light detection and ranging (LiDAR) information. The consequent shadows in these images are much darker and larger than those of pedestrian in our research. Therefore, they used the simple method of shadow detection which selected the area whose brightness was lower than pre-determined threshold. This method causes lots of error for shadow detection in our experimental images because the brightness of pedestrian is lower than that of shadow in many cases of our experimental images. Therefore, this method was not compared in our experiment.

As additional comparison, the method of [3] was also evaluated. The research [3] proposes the method of shadow detection based on foreground detection, vertical histogram analysis, foreground partitioning, calculation of the orientation of major axis, and decision. However, this method has the assumption that the position of light source should be known in advance. In addition, the authors assume that the case that the light source exists at the upper position of pedestrian (which can make the shadow at the lower position of the pedestrian) does not happen. However, in the outdoor at noon, this can occur frequently. Because the self-collected database in [3] is not available as open dataset, we applied their method to our database and CAVIAR open database which were used in our experiments. Following the 1st assumption of this method, we used the position of light source for experiment, which was manually labelled in each image frame.

As shown in Table 7, the accuracies by previous methods including the method [3] are lower than those by our method.

**Figure 8:**
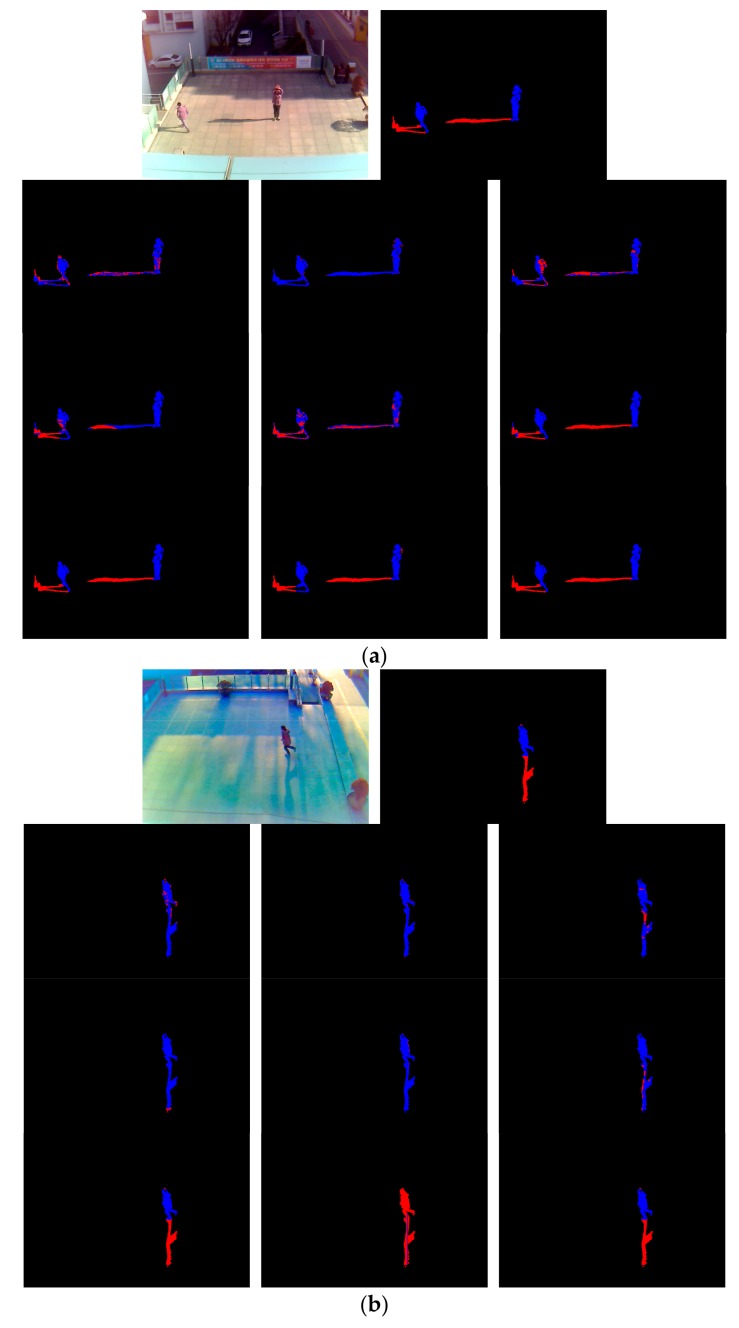
Detection results in experimental images: (**a**) Sub-database 1; (**b**) Sub-database 3. From (**a**) and (**b**), 10 images of each group are, from left to right: input image and ground truth image in the first row; the resultant images of Cucchiara et al. [8], Sanin et al. [15], and Leone et al. [17] in the second row; the resultant images of Hsieh et al. [19], Huang et al. [20], and the Euclidean distance by 4096 features of the first FCL [41] in the third row; and the resultant images of AlexNet [27], Lee et al. [3], and the proposed method in the fourth row. Non-shadow and shadow regions are shown as blue and red colors, respectively.

Figure 8 shows detected images of the proposed method and other compared methods along with the ground truth. In Figure 8a,b, 10 images of each group are, from left to right: input image and ground truth image in the first row; the result images of Cucchiara et al. [8], Sanin et al. [15] and Leone et al. [17] in the second row; the result images of Hsieh et al. [19], Huang et al. [20], Euclidean distance by 4096 features of first FCL [41] in the third row; and the resultant images of AlexNet [27], Lee et al. [3], and the proposed method in the fourth row.

As shown in Figure 8, the method of Cucchiara et al. [8] cannot discriminate shadow and non-shadow pixels, in most cases. The method of Sanin et al. [15] shows good results, showing light shadows. However, it does not show good results when a shadow is dark and similar to an object. Leone et al. [17] proposed a method showing better detection performance than the method by Cucchiara et al. [8], but still produces frequent errors. The method of Hsieh et al. [19], as shown in Figure 8a, produces good detection performance when a shadow cast beside a man, but degrades when the shadow is cast under a man, as shown in Figure 8b. The method of Huang et al. [20] shows better detection performance than using only color information, but still does not produce a good result. The Euclidean distance by 4096 features of the first FCL [41] also contains detection errors. This is because, although optimal features are obtained by CNN, the Euclidean distance-based linear classifier is used without the FCL nonlinear classifier of the third column of Table 2, which increases the detection errors. In the case where AlexNet [27] is used, the result image is close to the ground truth image and the result image of the proposed method. However, as shown in Table 7, the proposed method has higher detection accuracy. The accuracy by [3] is lower than our method as shown in Figure 8. That is because in their method [3], the separation of shadow region from pedestrian is done only by vertical line, and accurate position of shadow pixel in various direction cannot be detected as shown in the second image of the 4th row ones of Figure 8a. In addition, the shadow at the lower position of the pedestrian cannot be detected as shown in the second image of the 4th row ones of Figure 8b.

### 4.4. Testing with Another Open Database

**Figure 9:**
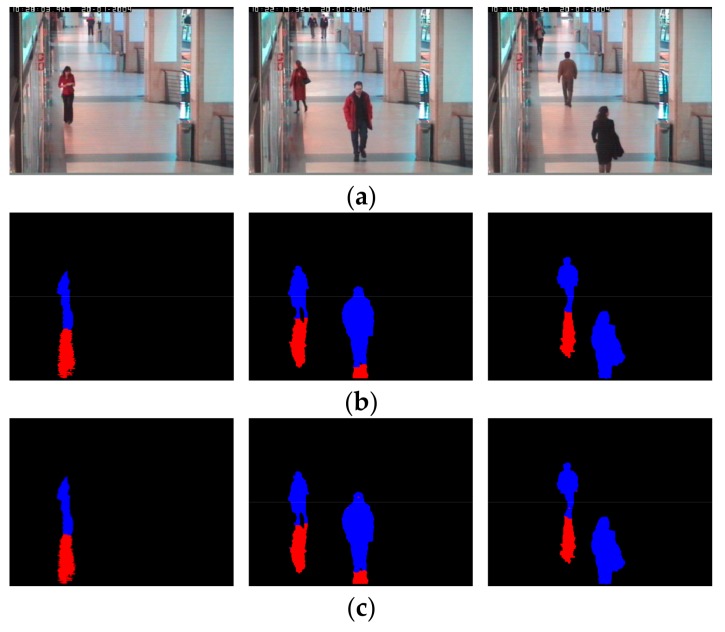
Experimental images using CAVIAR dataset: (**a**) Input images; (**b**) Ground truth images; and (**c**) Result images by our method.

We conduct another performance evaluation by applying the context aware vision using image-based active recognition (CAVIAR) open dataset [42]. The experiment is conducted as two-fold cross validation the same way as with DSDD-DB1 in Section 4.2 and Section 4.3. Figure 9 contains the result images obtained by experimenting with the CAVIAR dataset using the proposed method. The first row is the input frame, the second and third rows are the ground truth and the result image detected by the proposed method, respectively, for each input frame of the corresponding column. As shown in Figure 9, the proposed method’s detection results are very close to the ground truth image. Consequently, it turns out that the proposed method is applicable to various data environments.

The next experiment compares the detection accuracy between the proposed method and the existing methods [2,15,17,19,20]. To use the shadow detection rate (i.e., TPR) and the shadow discrimination rate (i.e., TNR), which are the metric of the existing researches [2,15] for comparing performance, this research also uses TPR and TNR as in Equations (6) and (10).

(10) $$\mathrm{TNR}\;=\frac{\#\mathrm{TN}}{\#\mathrm{TN}\;+\;\#\mathrm{FP}}$$

As Table 8 indicates, the mean accuracy of the proposed method is 97.67%, which is higher than those of the existing methods [2,3,15,17,19,20]. The most of shadows exist at the lower position of pedestrian in CAVIAR open database as shown in Figure 9. Therefore, the most pixels of pedestrian were incorrectly identified as shadow (FP case), which increased #FP and the consequent TNR of Equation (10) was decreased as shown in Table 8.

## 5. Conclusions

This research proposed a shadow detection and removal method that uses 21 × 21 sliding window-based VGG Net-16 CNN and showed a high accuracy, even in a high-definition surveillance condition. The experiments were conducted with an open database and our own database, collected at various times of day (i.e., morning, afternoon, and evening) under diverse weather, temperature, and illumination conditions. The proposed method’s robustness was demonstrated through various environmental changes. The proposed method was also compared to many of the existing methods. Our method had higher accuracy. Additionally, we opened the CNN model trained in this research and our own experimental database [7] so that other researchers can conduct fair comparisons.

A stronger network such as ResNet or DenseNet can enhance the accuracy of shadow classification. However, they have lots of additional interconnections (short-cuts) with parameters, which can increase processing time. In addition, as shown in Table 7 and Table 8, the accuracies by our shallow CNN-based method are sufficiently higher than 95% and 97%, respectively. Therefore, considering both the processing speed and accuracy, we used a shallower CNN of VGG Net-16 than ResNet or DenseNet which has deeper and stronger networks.

Our future research will consider a method of using various types of CNN, such as a semantic segmentation network to detect shadow and non-shadow regions directly from the entire input image without background subtraction. We also will examine a detection method considering information in continuous images and a method of classifying shadow and non-shadow pixels by combining CNN features and hand-crafted features.

## Figures and Tables

**Table 1:** Comparisons of previous and proposed research on shadow detection.

Category	Methods	Strength	Weakness
Non-learning-based method	HSV [8]	Shorter processing time through color information-centered shadow detection	Because only color information or gray texture are used, this method is susceptible to changes in object and illuminators which have similar color and texture to shadow and is little applicable to real world conditions including many variables.
	RGB [9]		
	YUV [13]		
	Normalized RGB [11,12]		
	*C*_1_*C*_2_*C*_3_ [10]		
	YCbCr [14]		
	Color + gradient information [15]	Additional information except color is used to improve the detection accuracy.	
	Luminance, chrominance, the difference in gradient density, and boundary characteristics of foreground [16]		
	Gabor texture feature [17]		
	Vertical histogram analysis and calculation of orientation considering the position of light source [3]	Shorter processing time	It uses the assumption that the position of light source should be known in advance and the light source should not exist at the upper position of pedestrian.
Learning-based method	GMM [18,20], Gaussian shadow model [19]	Better shadow detection performance than non-learning-based method, by the learning of various features	Optimal hand-craft feature cannot be manually extracted, and the application of the method is restricted to a specific type of environment. Thus, it can hardly be applied to various environments.
	HMM [21]		
	Neural-fuzzy system [22]		
	PCA + GMM [23]		
	SVM [24]		
	CNN (proposed method)	- Because massive data are learned, good performance of shadow detection can be obtained in various environments. - Optimal features are automatically extracted in learning, which does not need manual extraction.	Time-consuming training is needed which uses massive data.

**Table 2:** Detail explanations of CNN configuration.

Layer Type		Number of Filters	Size of Feature Map (Height × Width × Channel)	Size of Filters	Number of Stride	Number of Padding
Image input Layer			224×224×3			
Group 1	1st convolutional layer	64	224×224×64	3×3×3	1×1	1×1
	ReLU layer		224×224×64			
	2nd convolutional layer	64	224×224×64	3×3×64	1×1	1×1
	ReLU layer		224×224×64			
	Max pooling layer	1	112×112×64	2×2	2×2	0×0
Group 2	3rd convolutional layer	128	112×112×128	3×3×64	1×1	1×1
	ReLU layer		112×112×128			
	4th convolutional layer	128	112×112×128	3×3×128	1×1	1×1
	ReLU layer		112×112×128			
	Max pooling layer	1	56×56×128	2×2	2×2	0×0
Group 3	5th convolutional layer	256	56×56×256	3×3×128	1×1	1×1
	ReLU layer		56×56×256			
	6th convolutional layer	256	56×56×256	3×3×256	1×1	1×1
	ReLU layer		56×56×256			
	7th convolutional layer	256	56×56×256	3×3×256	1×1	1×1
	ReLU layer		56×56×256			
	Max pooling layer	1	28×28×256	2×2	2×2	0×0
Group 4	8th convolutional layer	512	28×28×512	3×3×256	1×1	1×1
	ReLU layer		28×28×512			
	9th convolutional layer	512	28×28×512	3×3×512	1×1	1×1
	ReLU layer		28×28×512			
	10th convolutional layer	512	28×28×512	3×3×512	1×1	1×1
	ReLU layer		28×28×512			
	Max pooling layer	1	14×14×512	2×2	2×2	0×0
Group 5	11th convolutional layer	512	14×14×512	3×3×512	1×1	1×1
	ReLU layer		14×14×512			
	12th convolutional layer	512	14×14×512	3×3×512	1×1	1×1
	ReLU layer		14×14×512			
	13th convolutional layer	512	14×14×512	3×3×512	1×1	1×1
	ReLU layer		14×14×512			
	Max pooling layer	1	7×7×512	2×2	2×2	0×0
	1st FCL		4096×1			
	ReLU layer		4096×1			
	Dropout layer		4096×1			
	2nd FCL		4096×1			
	ReLU layer		4096×1			
	Dropout layer		4096×1			
	3rd FCL		2×1			
	SoftMax layer		2×1			
	Classification layer		2×1			

**Table 3:** Description of five datasets.

Dataset	Condition	Detail Description
I (see Figure 4a)	−0.9 °C, afternoon, sunny, humidity 24%, wind 3.6 m/s	- Shadow with dark color cast due to strong sunlight.
II (see Figure 4b)	−6.0 °C, afternoon, cloudy, humidity 39%, wind 1.9 m/s	- Sunlight weakened by cloud so that a shadow of lighter color than in Figure 4a is cast.
III (see Figure 4c)	8.0 °C, evening, cloudy, humidity 42%, wind 3.5 m/s	- Darker image due to weak evening sunlight. - Long and many shadows due to the sun position in the evening and the reflection on buildings.
IV (see Figure 4d)	−5.2 °C, morning, sunny humidity 37%, wind 0.6 m/s	- Background and object become less distinguishable due to strong morning sunlight.
V (see Figure 4e)	13.8 °C, afternoon, rainy, humidity 65%, wind 2.0 m/s	- Overall dark image due to rainy day. - Many shadows generated by wet background floor.

**Table 4:** Number of data for 2-fold cross validation in our experiments.

	Non-Shadow	Shadow
**Group 1**	347,617	156,348
**Group 2**	349,075	155,214
**Total**	696,692	311,562

**Table 5:** The confusion matrix of testing results (unit: %).

Testing		Predicted	
		Shadow	Non-Shadow
Actual (Testing 1)	Shadow	94.86	5.14
	Non-shadow	1.60	98.40
Actual (Testing 2)	Shadow	95.47	4.53
	Non-shadow	1.74	98.26
Actual (Average)	Shadow	95.17	4.83
	Non-shadow	1.67	98.33

**Table 6:** Accuracies of shadow detection by our method (unit: %).

	TPR	PPV	ACC	F1_score
Testing 1	94.86	96.34	97.31	95.59
Testing 2	95.47	96.10	97.39	95.79
Average	95.17	96.22	97.35	95.69

**Table 7:** Comparisons of accuracy of classification by our method to previous methods (unit: %).

Methods	TPR	PPV	ACC	F1_Score
Cucchiara et al. [8]	19.98	30.96	61.51	24.29
Sanin et al. [15]	17.95	57.25	70.51	27.33
Leone et al. [17]	44.07	48.89	68.46	46.36
Hsieh et al. [19]	63.39	52.45	70.94	57.41
Huang et al. [20]	47.19	50.40	69.34	48.74
Euclidean distance by 4096 features of first FCL [41]	84.67	91.26	92.76	87.84
AlexNet [27]	94.18	95.59	96.86	94.88
Lee et al. [3]	81.49	43.48	61.35	56.70
Our method	95.17	96.22	97.35	95.69

**Table 8:** Comparison of accuracy for CAVIAR dataset (unit: %).

Methods	TPR	TNR	Average
Sanin et al. [2] (Chromaticity-based method)	92 *	56 *	74
Sanin et al. [15]	92.05	97.85	94.95
Leone et al. [17]	72 *	83 *	77.5
Hsieh et al. [19]	54 *	65 *	59.5
Huang et al. [20]	79 *	75 *	77
AlexNet [27]	97.09	97.15	97.12
Lee et al. [3]	77.53	21.08	49.3
Our method	97.96	97.38	97.67

* approximate value reported in [2].
